# 17-Hy­droxy-1,8-dimethyl-17-aza­penta­cyclo­[6.6.5.0^2,7^.0^9,14^.0^15,19^]nona­deca-2,4,6,9(14),10,12-hexa­ene-16,18-dione

**DOI:** 10.1107/S1600536812045151

**Published:** 2012-11-07

**Authors:** Barbara Miroslaw, Anna E. Koziol, Magdalena Pakosinska-Parys, Marta Struga

**Affiliations:** a Faculty of Chemistry, Maria Curie-Sklodowska University, pl. M. Curie-Sklodowskiej 3, 20-031 Lublin, Poland; b Department of Medical Chemistry, The Medical University, 02-007 Warsaw, Poland

## Abstract

In the title compound, C_20_H_17_NO_3_ (alternative name: *N*-hy­droxy-9,10-dimethyl-9,10-ethano­anthracene-11,12-dicarboximide), the rigid ethano­anthracene-dicarboximide moiety has a roof-shaped geometry, the inter­planar angle between the two terminal phenyl rings being 124.9 (6)°. In the crystal, mol­ecules are linked *via* O—H⋯O hydrogen bonds, forming chains along [010]. C—H⋯O and C—H⋯π inter­actions link adjacent chains, leading to the formation of a three-dimensional structure.

## Related literature

For the synthesis of the title compound, see: Kossakowski & Jarocka (2000 ▶). For the biological activity of related compounds, see: Bova *et al.* (2009 ▶). For related structures, see: Atherton & Jones (2002 ▶); Smet *et al.* (2000 ▶); Su *et al.* (2011 ▶), Guo *et al.* (2010 ▶); Adams *et al.* (2006 ▶); He & Ng (2007 ▶); Weber *et al.* (1991 ▶, 1994 ▶); Yang & Swager (1998 ▶). The rigid ethano­anthracenedicarboximide moiety of the title compound shows the typical roof-shaped geometry (Weber *et al.*, 1991 ▶; Csöregh *et al.*, 2003 ▶). For a description of the Cambridge Structural Database, see: Allen (2002 ▶).
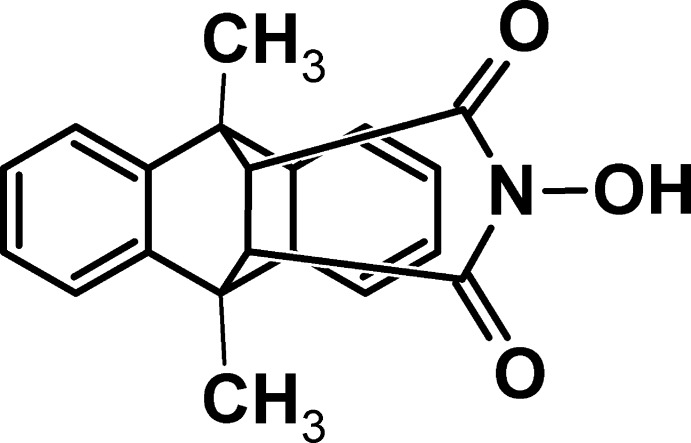


## Experimental

### 

#### Crystal data

C_20_H_17_NO_3_
*M* *_r_* = 319.36
Monoclinic, 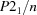

*a* = 13.904 (1) Å
*b* = 8.104 (1) Å
*c* = 13.946 (1) Å
β = 97.39 (1)°
*V* = 1558.4 (3) Å^3^
*Z* = 4
Mo *K*α radiation
μ = 0.09 mm^−1^
*T* = 100 K
0.40 × 0.40 × 0.30 mm

#### Data collection

Oxford Diffraction Xcalibur (Sapphire2) diffractometer
5321 measured reflections
2827 independent reflections
2467 reflections with *I* > 2σ(*I*)
*R*_int_ = 0.023

#### Refinement

*R*[*F* ^2^ > 2σ(*F* ^2^)] = 0.036
*wR*(*F* ^2^) = 0.096
*S* = 1.04
2827 reflections
223 parameters
H atoms treated by a mixture of independent and constrained refinement
Δρ_max_ = 0.30 e Å^−3^
Δρ_min_ = −0.20 e Å^−3^

### 

Data collection: *CrysAlis PRO* (Oxford Diffraction, 2009 ▶); cell refinement: *CrysAlis PRO*; data reduction: *CrysAlis PRO*; program(s) used to solve structure: *SHELXS97* (Sheldrick, 2008 ▶); program(s) used to refine structure: *SHELXL97* (Sheldrick, 2008 ▶); molecular graphics: *ORTEP-3* (Farrugia, 1997 ▶); software used to prepare material for publication: *publCIF*.

## Supplementary Material

Crystal structure: contains datablock(s) I, global. DOI: 10.1107/S1600536812045151/bg2475sup1.cif

Supplementary material file. DOI: 10.1107/S1600536812045151/bg2475Isup2.mol

Structure factors: contains datablock(s) I. DOI: 10.1107/S1600536812045151/bg2475Isup3.hkl

Supplementary material file. DOI: 10.1107/S1600536812045151/bg2475Isup4.cml

Additional supplementary materials:  crystallographic information; 3D view; checkCIF report

## Figures and Tables

**Table 1:** Hydrogen-bond geometry (Å, °) *Cg*1 is the centroid of the C6–C11 ring. *Cg*2 refers to the mid-point of the C15—C16 bond.

*D*—H⋯*A*	*D*—H	H⋯*A*	*D*⋯*A*	*D*—H⋯*A*
O3—H3*A*⋯O1^i^	0.96 (2)	1.69 (2)	2.630 (1)	167 (2)
C8—H8⋯O1^ii^	0.95	2.54	3.408 (2)	152
C15—H15⋯O3^iii^	0.95	2.46	3.273 (2)	143
C17—H17⋯O2^iv^	0.95	2.54	3.457 (2)	163
C4—H4⋯*Cg*1^v^	1.00	2.66	3.518 (3)	144
C10—H10⋯*Cg*2^vi^	0.95	2.77	3.668 (3)	158

Symmetry codes: (i) 
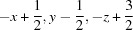
; (ii) 
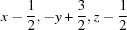
; (iii) 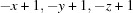; (iv) 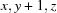; (v) 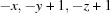; (vi) 
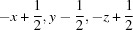
.

**Fig. 1.**
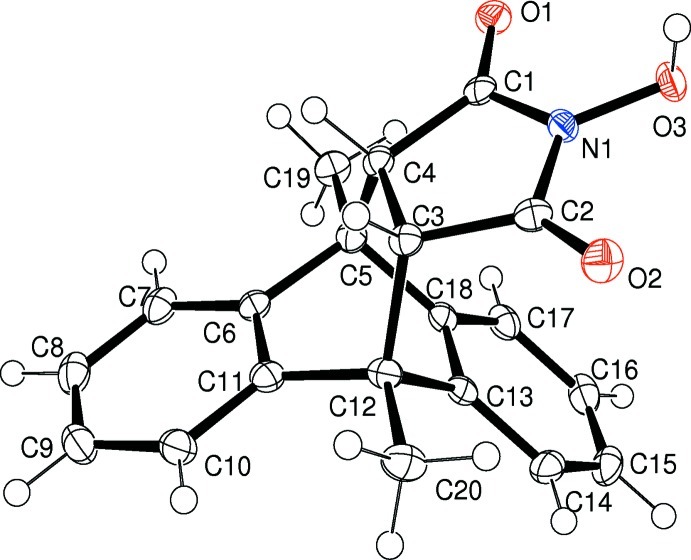
Ortep view of the title compound with atom numbering scheme. Ellipsoids for non-hydrogen atoms were drawn at the 50 % probability level.

**Fig. 2.**
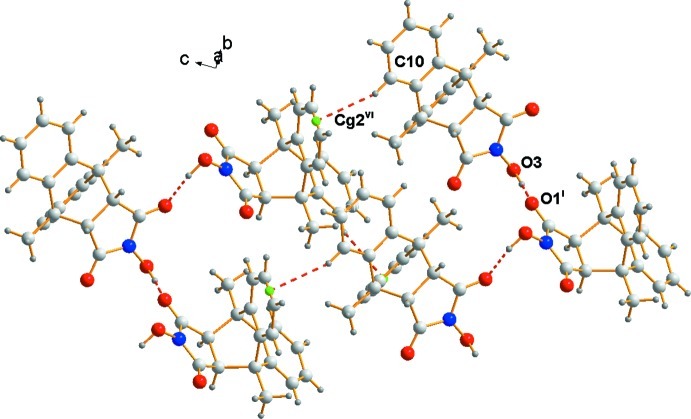
Linear association of molecules in crystal of 1 through the O–H···O hydrogen bonds and C–H···π interactions between adjacent chains. The Cg2 refers to the center of gravity between atoms C15/C16. Symmetry codes: (i) –x+1/2, y–1/2, –z+3/2; (vi) –x+1/2, y–1/2, –z+1/2.

**Fig. 3.**
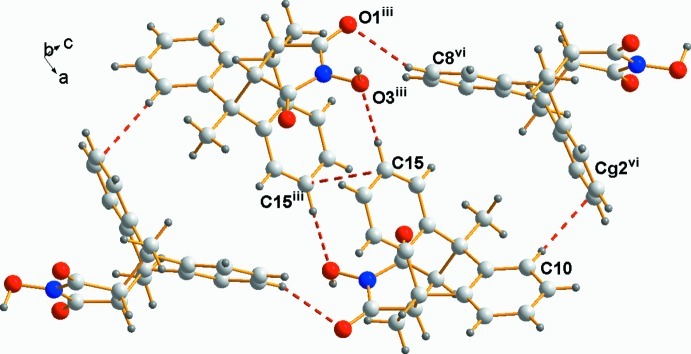
C–H···O and C–H···π interactions in crystal of 1 with short C15···C15iii intermolecular contact (3.386 (2) Å). The Cg2 is the centroid of the C15/C16 bond. Symmetry codes: (iii) –x+1, –y+1, –z+1; (vi) –x+1/2, y–1/2, –z+1/2.

**Fig. 4.**
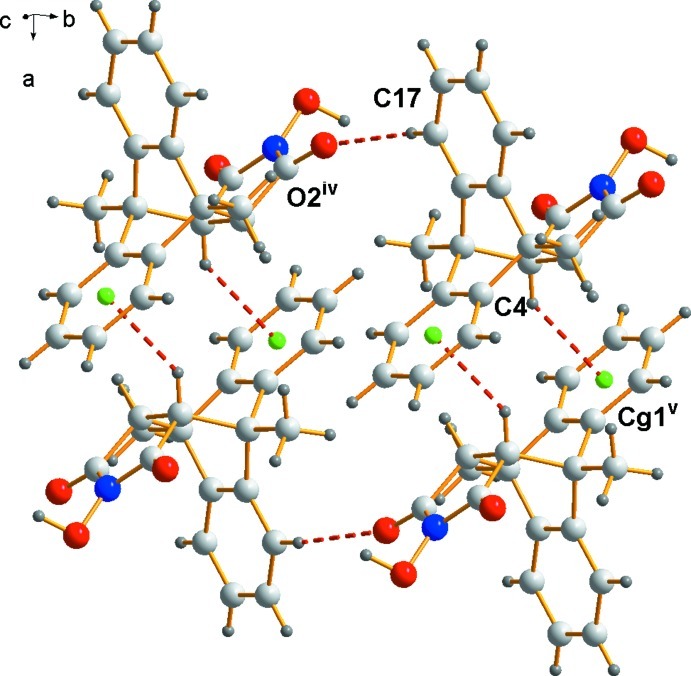
C–H···O hydrogen bonds and C–H···π interactions in crystal of 1. The Cg1 refers to the centroid of the C6–C11 ring. Symmetry codes: (iv) x, y+1, z; (v) –x, –y+1, –z+1.

## supplementary crystallographic information

### Comment

Roof-shaped aromatic hydrocarbon derivatives have been used for inclusion of neutral compounds (Weber *et al.*, 1991; 1994) and as porous polymer films and sensors for dinitrotoluene with possible application as a land mine detectors (Yang & Swager, 1998). The *N*-substitution is the most common way of modification of these molecules (Weber *et al.*, 1994; Smet *et al.*, 2000). Some experiments were also conducted on the substitution in the aryl moiety (Atherton & Jones, 2002; Adams *et al.*, 2006; He & Ng, 2007). The search of the CSD (CSD v5.33 and updates; Allen, 2002) revealed 67 crystal structures of compounds with the rigid pentacyclic 9,10-ethanoanthracenedicarboximide skeleton. However, none of these derivatives has *N*-hydroxy substituent and only two molecules are symmetrically substituted at bridgehead C atoms (here C5, C12; see Fig. 1). In these crystals the polycyclic skeletons are combined with voluminous macrocyclic fragments. (Su *et al.*, 2011; Guo *et al.*, 2010). The rigid ethanoanthracenedicarboximide moiety of the title compound (Fig. 1) shows the typical roof-shaped geometry (Weber *et al.*, 1991; Csöregh *et al.*, 2003); the interplanar angle between the two terminal phenyl rings is 124.9 °. The hydroxyl O atom interacts through the O3–H···O1 hydrogen bond (Fig. 2). Molecules form chains along the 2_1_ screw axis. Between adjacent chains many C–H···O and C–H···*π* interactions are observed (Figs. 2–4, Table 1).

### Experimental

The title compound, UPAC name: 17-hydroxy-1,8-dimethyl-17-azapentacyclo[6.6.5.0^2,7^.0^9,14^.0^15,19^]nonadeca-2,4,6,9(14),10,12-hexaene-16,18-dione, was synthesized in the search of compounds with potential anxiolytic activity, as described previously (Kossakowski & Jarocka, 2000).

### Refinement

All C-bonded H atoms were positioned geometrically and allowed to ride on the attached atom with the C—H bond lengths of 0.95 Å for aromatic atoms, 1.00 Å for methine and 0.98 Å for methyl groups. *U*_iso_(H) values were fixed to 1.2*U*_eq_(C) and 1.5*U*_eq_(C_methyl_). The hydroxyl H atom was located in the difference electron density map and refined isotropically.

### Crystal data

**Table:**

C_20_H_17_NO_3_	*F*(000) = 672
*M**_r_* = 319.36	*D*_x_ = 1.361 Mg m^−^^3^
Monoclinic, *P*2_1_/*n*	Mo *K*α radiation, λ = 0.71073 Å
Hall symbol: -P 2yn	Cell parameters from 3666 reflections
*a* = 13.904 (1) Å	θ = 2.9–29.8°
*b* = 8.104 (1) Å	µ = 0.09 mm^−^^1^
*c* = 13.946 (1) Å	*T* = 100 K
β = 97.39 (1)°	Prism, colourless
*V* = 1558.4 (3) Å^3^	0.40 × 0.40 × 0.30 mm
*Z* = 4	

### Data collection

**Table:**

Oxford Diffraction Xcalibur (Sapphire2) diffractometer	2467 reflections with *I* > 2σ(*I*)
Radiation source: Enhance (Mo) X-ray Source	*R*_int_ = 0.023
Graphite monochromator	θ_max_ = 25.2°, θ_min_ = 2.9°
Detector resolution: 8.4221 pixels mm^-1^	*h* = −16→16
ω scans	*k* = −9→6
5321 measured reflections	*l* = −9→16
2827 independent reflections	

### Refinement

**Table:**

Refinement on *F*^2^	Primary atom site location: structure-invariant direct methods
Least-squares matrix: full	Secondary atom site location: difference Fourier map
*R*[*F*^2^ > 2σ(*F*^2^)] = 0.036	Hydrogen site location: inferred from neighbouring sites
*wR*(*F*^2^) = 0.096	H atoms treated by a mixture of independent and constrained refinement
*S* = 1.04	*w* = 1/[σ^2^(*F*_o_^2^) + (0.0465*P*)^2^ + 0.4721*P*] where *P* = (*F*_o_^2^ + 2*F*_c_^2^)/3
2827 reflections	(Δ/σ)_max_ = 0.009
223 parameters	Δρ_max_ = 0.30 e Å^−^^3^
0 restraints	Δρ_min_ = −0.20 e Å^−^^3^

### Special details

**Table:**

Geometry. All s.u.'s (except the s.u. in the dihedral angle between two l.s. planes) are estimated using the full covariance matrix. The cell s.u.'s are taken into account individually in the estimation of s.u.'s in distances, angles and torsion angles; correlations between s.u.'s in cell parameters are only used when they are defined by crystal symmetry. An approximate (isotropic) treatment of cell s.u.'s is used for estimating s.u.'s involving l.s. planes.

### Fractional atomic coordinates and isotropic or equivalent isotropic displacement parameters (Å^2^)

**Table:**

	*x*	*y*	*z*	*U*_iso_*/*U*_eq_	
N1	0.28230 (8)	0.37858 (13)	0.63969 (8)	0.0154 (3)	
O1	0.23524 (7)	0.61166 (12)	0.71225 (6)	0.0201 (2)	
O2	0.30265 (7)	0.15262 (11)	0.54480 (7)	0.0217 (2)	
O3	0.36001 (7)	0.34569 (12)	0.70937 (7)	0.0194 (2)	
H3A	0.3338 (15)	0.258 (3)	0.7443 (15)	0.049 (6)*	
C1	0.22513 (9)	0.51497 (16)	0.64443 (9)	0.0148 (3)	
C2	0.25875 (10)	0.27735 (16)	0.55890 (9)	0.0161 (3)	
C3	0.17056 (9)	0.35563 (16)	0.49956 (9)	0.0151 (3)	
H3	0.1129	0.2818	0.4999	0.018*	
C4	0.15350 (9)	0.51968 (15)	0.55294 (9)	0.0147 (3)	
H4	0.0859	0.5217	0.5702	0.018*	
C5	0.16945 (10)	0.67321 (16)	0.48659 (9)	0.0157 (3)	
C6	0.09511 (9)	0.64834 (16)	0.39688 (9)	0.0161 (3)	
C7	0.02301 (10)	0.76017 (17)	0.36135 (10)	0.0199 (3)	
H7	0.0172	0.8623	0.3936	0.024*	
C8	−0.04079 (10)	0.72243 (19)	0.27836 (11)	0.0242 (3)	
H8	−0.0894	0.7994	0.2538	0.029*	
C9	−0.03310 (10)	0.5725 (2)	0.23190 (10)	0.0252 (3)	
H9	−0.0771	0.5464	0.1761	0.030*	
C10	0.03931 (10)	0.45948 (19)	0.26700 (10)	0.0216 (3)	
H10	0.0442	0.3568	0.2351	0.026*	
C11	0.10405 (9)	0.49792 (17)	0.34867 (9)	0.0172 (3)	
C12	0.18898 (10)	0.38987 (16)	0.39219 (9)	0.0167 (3)	
C13	0.27923 (10)	0.49978 (16)	0.40301 (9)	0.0159 (3)	
C14	0.36774 (10)	0.45997 (18)	0.37202 (10)	0.0206 (3)	
H14	0.3746	0.3601	0.3379	0.025*	
C15	0.44605 (10)	0.56721 (19)	0.39128 (10)	0.0253 (3)	
H15	0.5065	0.5400	0.3704	0.030*	
C16	0.43639 (10)	0.71368 (19)	0.44081 (10)	0.0239 (3)	
H16	0.4903	0.7861	0.4534	0.029*	
C17	0.34813 (10)	0.75541 (17)	0.47223 (10)	0.0195 (3)	
H17	0.3417	0.8559	0.5059	0.023*	
C18	0.26956 (10)	0.64801 (16)	0.45364 (9)	0.0159 (3)	
C19	0.15742 (11)	0.83661 (16)	0.53834 (10)	0.0207 (3)	
H19A	0.0939	0.8391	0.5618	0.031*	
H19B	0.1619	0.9282	0.4932	0.031*	
H19C	0.2087	0.8474	0.5932	0.031*	
C20	0.19871 (11)	0.22982 (17)	0.33594 (10)	0.0230 (3)	
H20A	0.2102	0.2562	0.2698	0.034*	
H20B	0.1389	0.1654	0.3342	0.034*	
H20C	0.2533	0.1654	0.3678	0.034*	

### Atomic displacement parameters (Å^2^)

**Table:**

	*U*^11^	*U*^22^	*U*^33^	*U*^12^	*U*^13^	*U*^23^
N1	0.0145 (6)	0.0179 (6)	0.0133 (5)	−0.0008 (4)	0.0005 (4)	0.0025 (4)
O1	0.0195 (5)	0.0236 (5)	0.0173 (5)	−0.0011 (4)	0.0024 (4)	−0.0065 (4)
O2	0.0259 (6)	0.0150 (5)	0.0245 (5)	0.0039 (4)	0.0045 (4)	0.0008 (4)
O3	0.0143 (5)	0.0250 (5)	0.0176 (5)	−0.0010 (4)	−0.0024 (4)	0.0058 (4)
C1	0.0129 (6)	0.0164 (7)	0.0159 (7)	−0.0034 (5)	0.0058 (5)	0.0010 (5)
C2	0.0182 (7)	0.0144 (7)	0.0167 (7)	−0.0036 (5)	0.0062 (5)	0.0021 (5)
C3	0.0149 (6)	0.0152 (7)	0.0154 (7)	−0.0028 (5)	0.0032 (5)	−0.0006 (5)
C4	0.0130 (6)	0.0160 (6)	0.0155 (7)	−0.0015 (5)	0.0031 (5)	−0.0010 (5)
C5	0.0154 (7)	0.0153 (7)	0.0166 (7)	−0.0006 (5)	0.0026 (5)	0.0010 (5)
C6	0.0141 (7)	0.0190 (7)	0.0158 (7)	−0.0024 (5)	0.0049 (5)	0.0024 (5)
C7	0.0173 (7)	0.0193 (7)	0.0242 (8)	0.0006 (5)	0.0067 (6)	0.0055 (6)
C8	0.0158 (7)	0.0310 (8)	0.0261 (8)	0.0008 (6)	0.0034 (6)	0.0126 (6)
C9	0.0193 (7)	0.0376 (9)	0.0177 (7)	−0.0065 (6)	−0.0011 (6)	0.0059 (6)
C10	0.0202 (7)	0.0280 (8)	0.0168 (7)	−0.0038 (6)	0.0026 (5)	−0.0007 (6)
C11	0.0167 (7)	0.0207 (7)	0.0149 (7)	−0.0026 (5)	0.0048 (5)	0.0019 (5)
C12	0.0187 (7)	0.0187 (7)	0.0130 (6)	−0.0008 (5)	0.0028 (5)	−0.0005 (5)
C13	0.0172 (7)	0.0194 (7)	0.0110 (6)	0.0013 (5)	0.0015 (5)	0.0048 (5)
C14	0.0218 (7)	0.0233 (7)	0.0179 (7)	0.0056 (6)	0.0072 (6)	0.0054 (6)
C15	0.0170 (7)	0.0350 (9)	0.0251 (8)	0.0061 (6)	0.0079 (6)	0.0138 (7)
C16	0.0159 (7)	0.0310 (8)	0.0242 (8)	−0.0048 (6)	0.0005 (6)	0.0118 (6)
C17	0.0198 (7)	0.0206 (7)	0.0175 (7)	−0.0030 (6)	−0.0003 (5)	0.0055 (6)
C18	0.0158 (7)	0.0189 (7)	0.0127 (6)	0.0011 (5)	0.0012 (5)	0.0051 (5)
C19	0.0229 (7)	0.0174 (7)	0.0223 (7)	0.0001 (6)	0.0046 (6)	−0.0011 (6)
C20	0.0292 (8)	0.0219 (7)	0.0181 (7)	0.0003 (6)	0.0039 (6)	−0.0034 (6)

### Geometric parameters (Å, º)

**Table:**

N1—C1	1.3679 (17)	C9—H9	0.9500
N1—O3	1.3831 (14)	C10—C11	1.3933 (19)
N1—C2	1.3981 (17)	C10—H10	0.9500
O1—C1	1.2223 (16)	C11—C12	1.5314 (19)
O2—C2	1.2099 (16)	C12—C13	1.5304 (19)
O3—H3A	0.96 (2)	C12—C20	1.5308 (19)
C1—C4	1.5149 (18)	C13—C14	1.3936 (19)
C2—C3	1.5264 (18)	C13—C18	1.4085 (18)
C3—C4	1.5567 (17)	C14—C15	1.392 (2)
C3—C12	1.5758 (18)	C14—H14	0.9500
C3—H3	1.0000	C15—C16	1.388 (2)
C4—C5	1.5831 (18)	C15—H15	0.9500
C4—H4	1.0000	C16—C17	1.397 (2)
C5—C19	1.5275 (18)	C16—H16	0.9500
C5—C6	1.5303 (18)	C17—C18	1.3948 (19)
C5—C18	1.5345 (18)	C17—H17	0.9500
C6—C7	1.3944 (19)	C19—H19A	0.9800
C6—C11	1.4053 (19)	C19—H19B	0.9800
C7—C8	1.399 (2)	C19—H19C	0.9800
C7—H7	0.9500	C20—H20A	0.9800
C8—C9	1.387 (2)	C20—H20B	0.9800
C8—H8	0.9500	C20—H20C	0.9800
C9—C10	1.402 (2)		
			
C1—N1—O3	121.87 (10)	C11—C10—H10	120.0
C1—N1—C2	115.83 (11)	C9—C10—H10	120.0
O3—N1—C2	122.27 (11)	C10—C11—C6	119.83 (12)
N1—O3—H3A	100.7 (12)	C10—C11—C12	125.48 (12)
O1—C1—N1	123.07 (12)	C6—C11—C12	114.68 (11)
O1—C1—C4	129.30 (12)	C13—C12—C20	114.73 (11)
N1—C1—C4	107.62 (11)	C13—C12—C11	106.67 (11)
O2—C2—N1	123.39 (12)	C20—C12—C11	113.32 (11)
O2—C2—C3	130.24 (12)	C13—C12—C3	103.89 (10)
N1—C2—C3	106.36 (11)	C20—C12—C3	111.94 (11)
C2—C3—C4	104.88 (10)	C11—C12—C3	105.43 (10)
C2—C3—C12	111.78 (11)	C14—C13—C18	119.86 (13)
C4—C3—C12	110.94 (10)	C14—C13—C12	125.47 (12)
C2—C3—H3	109.7	C18—C13—C12	114.58 (11)
C4—C3—H3	109.7	C15—C14—C13	119.71 (13)
C12—C3—H3	109.7	C15—C14—H14	120.1
C1—C4—C3	104.90 (10)	C13—C14—H14	120.1
C1—C4—C5	112.69 (10)	C16—C15—C14	120.43 (13)
C3—C4—C5	110.51 (10)	C16—C15—H15	119.8
C1—C4—H4	109.5	C14—C15—H15	119.8
C3—C4—H4	109.5	C15—C16—C17	120.54 (13)
C5—C4—H4	109.5	C15—C16—H16	119.7
C19—C5—C6	113.39 (11)	C17—C16—H16	119.7
C19—C5—C18	114.55 (11)	C18—C17—C16	119.30 (13)
C6—C5—C18	106.32 (10)	C18—C17—H17	120.3
C19—C5—C4	111.91 (10)	C16—C17—H17	120.3
C6—C5—C4	104.10 (10)	C17—C18—C13	120.16 (12)
C18—C5—C4	105.72 (10)	C17—C18—C5	125.47 (12)
C7—C6—C11	119.84 (12)	C13—C18—C5	114.35 (11)
C7—C6—C5	125.74 (12)	C5—C19—H19A	109.5
C11—C6—C5	114.42 (11)	C5—C19—H19B	109.5
C6—C7—C8	120.17 (13)	H19A—C19—H19B	109.5
C6—C7—H7	119.9	C5—C19—H19C	109.5
C8—C7—H7	119.9	H19A—C19—H19C	109.5
C9—C8—C7	119.95 (13)	H19B—C19—H19C	109.5
C9—C8—H8	120.0	C12—C20—H20A	109.5
C7—C8—H8	120.0	C12—C20—H20B	109.5
C8—C9—C10	120.27 (13)	H20A—C20—H20B	109.5
C8—C9—H9	119.9	C12—C20—H20C	109.5
C10—C9—H9	119.9	H20A—C20—H20C	109.5
C11—C10—C9	119.92 (14)	H20B—C20—H20C	109.5

### Hydrogen-bond geometry (Å, º)

Cg1 is the centroid of the C6–C11 ring. Cg2 refers to the mid-point of the C15—C16 bond.

**Table:**

*D*—H···*A*	*D*—H	H···*A*	*D*···*A*	*D*—H···*A*
O3—H3*A*···O1^i^	0.96 (2)	1.69 (2)	2.630 (1)	167 (2)
C8—H8···O1^ii^	0.95	2.54	3.408 (2)	152
C15—H15···O3^iii^	0.95	2.46	3.273 (2)	143
C17—H17···O2^iv^	0.95	2.54	3.457 (2)	163
C4—H4···*Cg*1^v^	1.00	2.66	3.518 (3)	144
C10—H10···*Cg*2^vi^	0.95	2.77	3.668 (3)	158

Symmetry codes: (i) −*x*+1/2, *y*−1/2, −*z*+3/2; (ii) *x*−1/2, −*y*+3/2, *z*−1/2; (iii) −*x*+1, −*y*+1, −*z*+1; (iv) *x*, *y*+1, *z*; (v) −*x*, −*y*+1, −*z*+1; (vi) −*x*+1/2, *y*−1/2, −*z*+1/2.
